# Differential Selection for Survival and for Growth in Adaptive Laboratory Evolution Experiments With Benzalkonium Chloride

**DOI:** 10.1111/eva.70017

**Published:** 2024-10-12

**Authors:** Selina B. I. Schmidt, Tom Täschner, Niclas Nordholt, Frank Schreiber

**Affiliations:** ^1^ Department of Materials and the Environment, Division of Biodeterioration and Reference Organisms (4.1) Federal Institute for Materials Research and Testing (BAM) Berlin Germany

**Keywords:** ALE experiments, Benzalkonium chloride, biocides, *cyaA*, disinfectants, *E. Coli*, *hslO*, preservatives, *tufA*

## Abstract

Biocides are used to control microorganisms across different applications, but emerging resistance may pose risks for those applications. Resistance to biocides has commonly been studied using adaptive laboratory evolution (ALE) experiments with growth at subinhibitory concentrations linked to serial subculturing. It has been shown recently that *Escherichia coli* adapts to repeated lethal stress imposed by the biocide benzalkonium chloride (BAC) by increased survival (i.e., tolerance) and not by evolving the ability to grow at increased concentrations (i.e., resistance). Here, we investigate the contributions of evolution for tolerance as opposed to resistance for the outcome of ALE experiments with *E. coli* exposed to BAC. We find that BAC concentrations close to the half maximal effective concentration (EC_50_, 4.36 μg mL^−1^) show initial killing (~40%) before the population resumes growth. This indicates that cells face a two‐fold selection pressure: for increased survival and for increased growth. To disentangle the effects of both selection pressures, we conducted two ALE experiments: (i) one with initial killing and continued stress close to the EC_50_ during growth and (ii) another with initial killing and no stress during growth. Phenotypic characterization of adapted populations showed that growth at higher BAC concentrations was only selected for when BAC was present during growth. Whole genome sequencing revealed distinct differences in mutated genes across treatments. Treatments selecting for survival‐only led to mutations in genes for metabolic regulation (*cyaA*) and cellular structure (flagella *fliJ*), while treatments selecting for growth and survival led to mutations in genes related to stress response (*hslO* and *tufA*). Our results demonstrate that serial subculture ALE experiments with an antimicrobial at subinhibitory concentrations can select for increased growth and survival. This finding has implications for the design of ALE experiments to assess resistance risks of antimicrobials in different scenarios such as disinfection, preservation, and environmental pollution.

## 1 Introduction

Antimicrobial resistance is a global burden and will likely become more severe in the future (Murray et al. 2021, 2022). While there is an ongoing increase of antimicrobial resistance, there is very little progress accomplished in the development and admission of new antimicrobial compounds (Butler et al. 2023; Butler and Paterson 2020; Prasad et al. 2022). Therefore, the efficacy of the available antimicrobial compounds must be preserved by preventing the evolution of antimicrobial resistance. This requires an understanding of the underlying mechanisms and causes for selection, evolution, and spread of antimicrobial resistance.

Biocides are used to control bacteria across various applications (Gnanadhas, Marathe, and Chakravortty 2013; Maillard 2005). This widespread use of biocides is reflected by the presence of biocide residues in the environment (Schoknecht et al. 2009). Biocides are clearly contributors to the burden of antimicrobial resistance (Jones and Joshi 2021; Kampf 2018; Kim et al. 2018; Russell 2003; Schmidt et al. 2022). A specific example for the consequences of using active substances used in biocidal products for antimicrobial resistance is the treatment failure in case of infections with human pathogenic fungi treated with azole‐based drugs in clinical settings. This treatment failure is partly related to the use of azole‐based pesticides or biocides, which contribute to the evolution and spread of azole resistant fungi in the environment (Berger et al. 2017; Fisher et al. 2018; Kleinkauf et al. 2013; Shelton et al. 2023; Verweij et al. 2020).

Benzalkonium chloride (BAC) is a quaternary ammonium compound used in a wide range of applications, for example, as an antiseptic, disinfectant, or as material preservative (Tezel and Pavlostathis 2015). This explains the occurrence of BAC pollution in various matrices like food, wastewater, soils, and sediments (Barber and Hartmann 2021; Mulder et al. 2018; Tezel, Pierson, and Pavlostathis 2006). Further on, low BAC concentrations which also occur in the environment can increase the spread of antibiotic resistance through horizontal gene transfer via transformation (Jia et al. 2023; Wang et al. 2023) or conjugation (Schmidt et al. 2022) and even promote the evolution of decreased susceptibility to antibiotics (Guérin et al. 2021; Soumet et al. 2016). Thus, the prolonged exposure of microorganisms to BAC contributes to the antimicrobial resistance crisis (Bore et al. 2007; Kim et al. 2018; Merchel Piovesan Pereira and Tagkopoulos 2019). While BAC resistance mechanisms and their effect on antimicrobial resistance evolution have been partly investigated, the underlying selective pressures during evolution under BAC exposure have received relatively little attention.

Adaptive laboratory evolution experiments (ALE) are commonly used to assess the risks and understand the mechanisms of resistance evolution to antibiotics, biocides, plant protection products, and antimicrobial peptides (Charron et al. 2023; Dragosits and Mattanovich 2013; Guérin et al. 2021; Jahn et al. 2017; Makarova et al. 2018; Pöppe et al. 2020; Van den Bergh et al. 2018; Webber et al. 2015). Therein, a serial transfer in batch, continuous culture or gradient agar plate of microorganisms in the presence of antimicrobials is used to study the population dynamics and molecular mechanisms of adaption (Baym et al. 2016; Jahn et al. 2017; Nordholt et al. 2021; Van den Bergh et al. 2018; Van Dijk et al. 2019). In typical serial transfer evolution experiments with antimicrobials, a bacterial batch culture is repeatedly diluted and grown in the presence of a subinhibitory concentration of the antimicrobial. It is assumed that the main selection pressure is for growth in the presence of the antimicrobial stress during the growth phase. However, little attention is paid to the initial population dynamics and whether a fraction of the population is killed upon exposure to the antimicrobial, as has been anecdotally observed for example for *Mycobacterium smegmatis* in response to the antibiotic isoniazid (Wakamoto et al. 2013). Such dynamics may exert a selection pressure for increased survival in addition to growth in the presence of the antimicrobial.

The initial population size could affect the initial population dynamics in ALE experiments by modulating the efficacy of an antimicrobial through a phenomenon termed “the inoculum effect” (Karslake et al. 2016). The inoculum effect has been observed with antibiotics (Brook 1989; Lenhard and Bulman 2019; Sabath et al. 1975), biocides (García and Cabo 2018; Haas and Kaymak 2003; Lambert and Johnston 2000), and antimicrobial peptides (Loffredo et al. 2021). The inoculum effect for certain antibiotics can cause an initial decrease of a fraction of the population while leaving the rest of the population potentially unaffected (Udekwu et al. 2009). Consequently, typical serial transfer evolution experiments may select not only for increased growth in the presence of antimicrobials but also for increased survival.

Mechanisms of microbes for increased survival are distinguished by three phenomena, termed resistance, tolerance, and persistence (Brauner et al. 2016). In the presence of an antimicrobial compound, resistant strains grow above the minimum inhibitory concentration (MIC) which suppresses growth of susceptible strains. Tolerant strains can survive exposure to lethal stress longer than susceptible strains, for instance by slower growth or longer lag phases (Brauner et al. 2016; Fridman et al. 2014). Persistence is related to a tolerant subpopulation that can survive a transient exposure to lethal stress while the majority of the population is killed (Brauner et al. 2016). Selection for increased survival from persistence can be achieved rapidly upon repeated exposure to lethal doses of antibiotics as well as biocides (Fridman et al. 2014; Nordholt et al. 2021; Van den Bergh et al. 2016). Tolerance and persistence promote the evolution (Levin‐Reisman et al. 2019; Windels et al. 2019) and spread of antibiotic resistance (Bakkeren et al. 2019; Levin and Rozen 2006). Moreover, resistance, tolerance, and persistence are underpinned by different molecular mechanisms, whereby high tolerance does not necessarily impose resistance (Brauner et al. 2016). The differences between these phenomena and their underlying mechanisms need to be considered when designing ALE experiments in order to use the outcomes for risk assessment.

**FIGURE 1:**
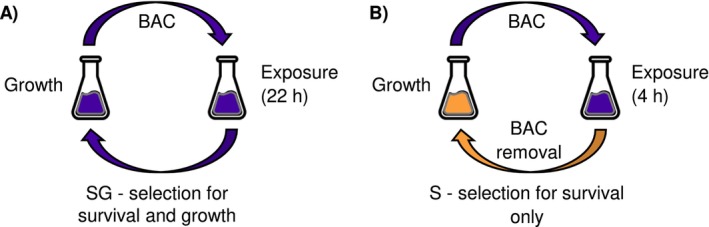
Serial subculture evolution experiment to assess distinct selection pressures of benzalkonium chloride (BAC). (A) Selection for survival and growth (SG) and (B) the selection for survival‐only (S). Each of the two evolutionary treatments consisted of 12 parallel populations of which 6 populations were exposed to BAC according to the evolutionary treatment and 6 additional populations served as controls treated without BAC exposure, but otherwise being propagated exactly in the same way as the BAC‐treated populations. One treatment cycle lasted for about 22 h before the culture was transferred to fresh medium. BAC was present for the entire duration, that is, during the lag and the growth phase, in the selection for survival and growth (SG) treatment, whereas BAC was removed after 4 h of exposure at the end of the lag phase (before the growth phase) in the selection for survival‐only (S) treatment.

The aim of this study was to disentangle the effects of different selection pressures being present in serial transfer ALE experiments with the biocide BAC. First, we determined the half‐maximal inhibitory concentration for growth (EC_50_) by establishing dose–response curves and performed time‐kill experiments at the EC_50_. The results showed that subinhibitory concentrations of BAC are lethal to a part of the population in the lag phase and thereby may exert selection for survival as well as selection for increased growth. To disentangle both selective drivers, we designed two serial transfer evolution experiments (Figure 1): (i) one treatment to select for survival and growth with BAC being present in the lag and growth phase (SG, Figure 1A), and (ii) a second treatment to select for survival‐only with BAC being present in the lag phase and removed before the growth phase (S, Figure 1B). Phenotypic and genotypic analysis of the evolved lines of both treatments indicate that selection for growth is not the only selection pressure in serial transfer evolution experiments with BAC. Our results emphasize that tolerance and resistance emerge via distinct mechanisms and that studying either phenomenon requires careful considerations and precise knowledge of the selection pressures during experimental evolution.

## 2 Material and Methods

### 2.1 Bacterial Strain, Chemicals, Media, and Cultivation Conditions

*Escherichia coli* K12 MG1655 was a generous gift from Prof. Rupert Mutzel (Freie Universität Berlin). The strain was preadapted to serial transfer in M9G medium for 150 generations as described previously (Nordholt et al. 2021). The strain was cultivated in liquid‐defined media M9G composed of M9 salts, minerals, trace elements, and glucose as source of carbon and energy (42.2 mM Na_2_HPO_4_, 22 mM KH_2_PO_4_, 11.3 mM (NH_4_)_2_SO_4_, 8.5 mM NaCl, 2 mM MgSO_4_, 0.45 mM CaCl_2_, 20 mM glucose, 0.0025 mM FeCl_3_, 0.00495 mM ZnCl_2_, 0.0021 mM CoCl_2_, 0.002 mM Na_2_MoO_4_, 0.0003 mM CaCl_2_, 0.0025 mM CuCl_2_, and 0.002 mM H_3_BO_3_) and incubated overnight at 220 rpm and 37°C. When cultivated on solid media, Lysogeny broth (LB) Lennox formulation (5 g L^−1^ yeast extract, 10 g L^−1^ tryptone, 5 g L^−1^ NaCl, and 15 g L^−1^ agar bacteriology grade) agar plates were used and incubated overnight at 37°C. Growth parameters of *E. coli*, such as growth rate and lag time, were determined using a plate reader (Biotek Epoch 2 microplate reader) set to 37°C, fast orbital shaking, reading optical density every 5 min. The lag times and growth rates were calculated from the increase in OD_600_ over time by inferring the time derivative using Gaussian processes implemented as published code (Swain et al. 2016) in Python 3.6. Benzalkonium chloride [BAC] (PO 12060) was purchased from (Merck KGaA, Darmstadt, Germany). The antibiotics colistin (PO 537340), gentamycin (PO 412367), ciprofloxacin (PO 412310), and ampicillin (PO 412252) were purchased as E‐test stripes from Biomérieux (bioMérieux Deutschland GmbH, Nürtingen, Germany).

### 2.2 Antimicrobial Susceptibility Determination

Susceptibility to BAC was determined with the broth microdilution method using 200 μL total volume per well (Andrews 2001) at the following concentrations 0; 3; 4; 6; 7; 8; 9; 10; 14; 15 μg mL^−1^. The minimum inhibition concentration (MIC) was defined as the lowest concentration that inhibits the growth in liquid cultures. The EC_50_ of BAC was determined by fitting the growth rate data at various BAC concentrations to a sigmoidal fit using a dose–response curve with variable Hill slope given by parameter “p” and the Levenberg Marquardt iterative algorithm in OriginLab (Origin 2021 Version 9.8.0.200).

The effect of the selected subinhibitory BAC concentration on the viable cell number of the *E. coli* ancestor and the evolved *E. coli* populations was assessed by performing growth curves, which were analyzed by plating and counting of colony forming units at certain time points. *E. coli* overnight cultures were adjusted to 10^6^ cfu mL^−1^ (cfu—colony forming units), incubated without and with 4 μg mL^−1^ BAC at 37°C and 1200 rpm in a tabletop incubator (Thermoshaker, Starlab). A subsample of the cell suspension was retrieved before the addition of BAC. Experiments were either performed by sampling at hourly intervals until 4 h or by sampling at a single timepoint after 2 h only. The subsample of the cell suspension was diluted appropriately, plated on LB agar, the plates were incubated at 37°C for 24 h, and the colony forming units were counted.

Susceptibility to antibiotics was assessed using E‐test stripes following the EUCAST disk diffusion guidelines (European Committee on Antimicrobial Susceptibility Testing (EUCAST) 2021) with slight modifications. Instead of MH2 agar plates, LB agar plates were used and incubated at 37°C. The *E. coli* ancestor was used as a reference point to the evolved *E. coli* populations with the two distinct BAC treatments.

### 2.3 Serial Subculture Evolution Experiment

Daily serial subculture evolution treatments were carried out over 15 days, resulting in approximately 150 generations. Each of the two evolutionary treatments (Figure 1), the selection for survival‐only and the selection for survival and growth treatment, had each six biological replicates treated with BAC and six corresponding controls treated without BAC, resulting in total 24 evolutionary populations. In both evolutionary treatments, BAC was present at sub‐MIC concentrations for 4 h. After 4 h of BAC exposure, the BAC‐containing M9G media was replaced by BAC‐free M9G media in the selection for survival‐only treatment, whereas the BAC‐containing M9G media remained in the selection for survival and growth treatment.

**FIGURE 2:**
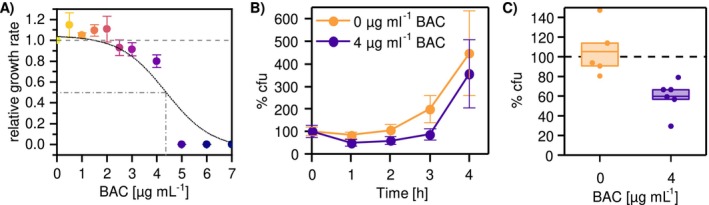
Benzalkonium chloride (BAC) susceptibility of the *E. coli* ancestor. (A) Dose–response curve for the growth rate of *E. coli* as a function of BAC concentration (color gradient from yellow to purple). The measured mean growth rate was calculated relative to the growth rate without exposure to BAC and displayed as circles with error bars denoting the standard deviation (*N* = 4 biological replicates). The black, dashed line shows the sigmoidal fit of a dose–response model and the gray, dashed‐dotted line depicts the EC_50_ equaling to 4.36 μg mL^−1^ BAC. (B) Initial kill and growth curve of *E. coli* with (purple line) and without (orange line) exposure to 4 μg mL^−1^ BAC displayed as % colony forming units (cfu) over time relative to the initial inoculum at time 0. Circles represent the mean with error bars denoting the standard deviation (*N* = 5 to 6 biological replicates). (C) The surviving fraction of *E. coli* after 2 h exposure relative to the initial inoculum at time 0 with and without 4 μg mL^−1^ BAC (data taken from the 2 h time point shown in panel B, note the different scaling of the y‐axis between panels B and C). The box displays the 25th and the 75th percentile, the center line displays the mean and the circles display individual biological replicates (*N* = 5–6). A statistically significant deviation from 100% was assessed for the treatment without BAC using a One‐Sample *t*‐test (*p* = 0.009; OriginLab, Origin 2021 Version 9.8.0.200).

All 12 cultures for the experimental treatments (i.e., selection for survival‐only and selection for survival and growth) were adjusted to 3 × 10^6^ cfu mL^−1^ in 520 μL fresh M9G media prior to exposure with 4 μg mL^−1^ BAC. The 12 additional cultures for the corresponding controls were exposed with fresh M9G medium only. The cell suspensions were incubated in a tabletop shaker (Thermoshaker, Starlab) at 37°C with 1200 rpm for 4 h and subsequently centrifuged for 5 min at 8000 rpm before the medium was changed to fresh M9G medium without BAC in the selection for survival treatment and their corresponding controls. To ensure equal treatment, the selection for survival and growth treatments were also centrifuged but the cell pellet was immediately resuspended in the supernatant (BAC‐containing M9G medium). We decided to not resuspend the survival and growth treatment in fresh BAC‐containing medium because BAC binds to the biomass (Nordholt et al. 2022), and thus adding additional BAC could have let to differences in exposure between both treatments. The additional possible genome replications for the survival and growth treatment as compared to the survival‐only treatment within the 4 h of exposure, mainly demarking the lag phase and the early onset of the growth phase (Figure 2B), are negligible.

Cultures of both treatments and the corresponding controls were incubated at 37°C and shaken in a tabletop (Thermoshaker, Starlab) at 1200 rpm for a total of 22 h (including the 4 h preincubation with BAC) to allow cell growth to stationary phase. The cell density at the end of a growth phase was ca. 3 × 10^9^ cfu mL^−1^. As the cultures were adjusted to 3 × 10^6^ cfu mL^−1^ prior to BAC exposure, the applied bottleneck during evolution is ca. 0.001. Daily freezer stocks were prepared in 20% glycerol and stored at −80°C. The evolved populations from the different treatments and controls from the last day were further used for phenotypic and genotypic characterization.

### 2.4 Genotypic Analysis of the Evolved Populations

Chromosomal DNA of the populations from four of six biological replicates of each treatment and from two of six replicates of the corresponding controls was isolated from glycerol freezer stocks using the peqGOLD bacterial DNA kit (PO 13‐3450‐01). Genome sequencing was performed by Eurofins Genomics (Eurofins Genomics Europe Sequencing GmbH, Konstanz, Germany) on an Illumina NovaSeq6000 system with 150 bp paired‐end reads and ~300‐fold coverage. Quality of the reads was assessed using fastqc (Wingett and Andrews 2018) showing that no further trimming was necessary. The analysis of mutations was performed using breseq (Deatherage and Barrick 2014) and the *E. coli* K12 MG1655 reference genome (NCBI RefSeq NC_000913.3). As described previously (Nordholt et al. 2021), there were four mutations in the ancestor compared with the reference genome NC_000913.3: Δ776 bp insB9–[crl], +8 bp bamD →/→ raiA, +GC gltP →/← yjcO, and a ~ 1800 bp inversion of the P‐element of prophage e14. Intra‐ and intergenic mutations were removed from the list of identified mutations of each BAC‐treated population based on the following two criteria: (i) mutations that were present below a threshold of 5% of the total reads, and (ii) mutations that were also present in the corresponding controls or in the ancestor strain. The mutated genes were clustered into categories by MultiFun Terms found on BioCyc (https://biocyc.org/). The sequences of populations evolved in this study where additionally compared to sequences from six populations that evolved for increased persistence upon exposure to lethal doses in a previous study and two corresponding control lineages (Nordholt et al. 2021). Population sequencing data from experiments described here and from those described in Nordholt et al. (2021) were deposited in sequence read archive (SRA) at NCBI under BioProject number PRJNA1074740. Illumina raw reads of the ancestor have been deposited earlier and can be accessed at SRA under the BioSample accession SAMN19555147.

## 3 Results

### 3.1 Subinhibitory BAC Concentrations Are Lethal for a Fraction of the Population and Delay Initial Growth

The aim of the study is to understand the drivers of selection during serial transfer evolution experiments with BAC. Common serial transfer evolution experiments operate with subinhibitory concentrations of antimicrobials to ensure growth and to simultaneously maintain a selection pressure. Therefore, the susceptibility of *E. coli* to BAC was tested, determining the dose–response curve and time‐kill kinetics. Dose–response analysis showed that BAC has a narrow concentration range (between 2.5 and 5 μg mL^−1^) at which it affects the growth rate (Figure 2A). The half maximal effective concentration (EC_50_), the concentration at which the growth rate is lowered to 50%, was estimated to be 4.36 μg mL^−1^ BAC. The MIC of BAC of the *E. coli* ancestor was determined to be 5 μg mL^−1^. Exposing *E. coli* to 4 μg mL^−1^ BAC showed that the initial growth was delayed as compared to unexposed cells (Figure 2B). The observed delay of growth was based on the significant reduction of 40% viable cells after 2 h in the presence of subinhibitory BAC concentrations (Figure 2C, One‐Sample *t*‐test *p* = 0.009). The data also indicate phenotypic heterogeneity in survival because only part of the cells are killed upon BAC exposure while other cells survive. These data suggest that serial transfer evolution experiments at subinhibitory concentrations may exert two types of selection pressures: selection for faster growth and enhanced survival at subinhibitory BAC concentrations.

### 3.2 Phenotypes Evolved in the Presence of BAC Are Linked to Selection Pressures

**FIGURE 3:**
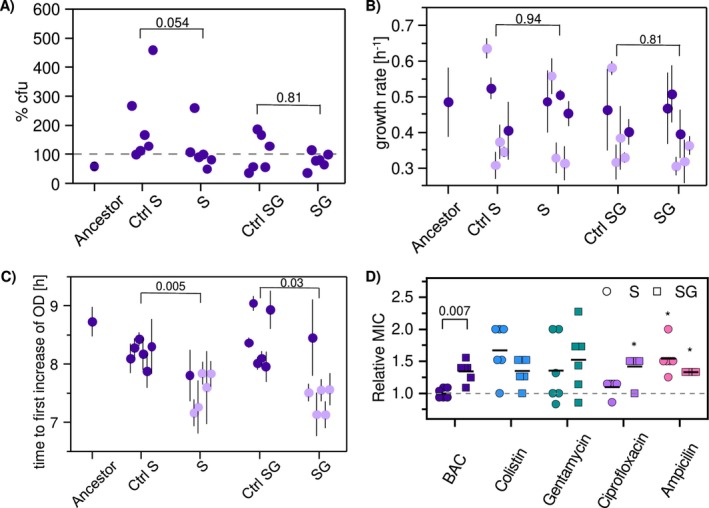
Phenotypic changes of *E. coli* populations evolved under selection for survival‐only (S) or survival and growth (SG) in the presence of BAC, their corresponding nonexposed controls (Ctrl) and the ancestor. (A) Surviving cell fraction in % colony forming units (cfu) after 2 h exposure with 4 μg mL^−1^ BAC relative to the beginning of the incubation. (B) Specific growth rates (h^−1^) and (C) time to first increase in optical density (OD in h) at 4 μg mL^−1^ BAC exposure. (D) Susceptibility to BAC and antibiotics as measured by the minimal inhibitory concentration (MIC) displayed relative to the MIC of the ancestor (dashed line). The raw MIC values are provided in Table S1. The two different evolutionary BAC treatments in panel D are indicated by circles for the survival‐only and squares for the survival and growth treatment. The tested antimicrobials can be distinguished by colors (dark blue, BAC; light blue, colistin; green, gentamycin; purple, ciprofloxacin; pink, ampicillin). For all panels, each data point represents one evolutionary lineage and the horizontal line indicates the mean. The error bars in (A) for the ancestor only, and in (B) and (C) for all datapoints indicate the standard deviation around the mean of biological replicates (*N* = 6 in A; *N* = 12 in B and C). Data in panels (B) and (C) were analyzed with One‐way ANOVA (panel B *p* = 1.7e^−86^; panel C *p* = 1.5e^−13^). Means of single evolved lineages shown in light purple color in panels (B) and (C) are significantly different to the ancestor (Bonferroni post hoc test, *p* < 0.05). If the values are shown in dark purple, there was no significant difference to the ancestor (*p* > 0.05), or a statistical test could not be performed due to the absence of replicate measurements for each single lineage (panel A). Significance between the evolutionary treatments and their corresponding controls is assessed with the Mann–Whitney *U* test. Exact *p* values are indicated above the brackets. Significant differences of antibiotics' MICs relative to the ancestor for all replicate populations in one evolutionary treatment in panel (D) are assessed with the One‐Sample Wilcoxon signed rank test against level 1 (*p < 0.05). All statistical tests were performed with OriginPro 2021 (OriginLab, Version 9.8.0.200).

To test whether subinhibitory BAC concentrations differentially affect evolution for survival or for survival and growth, we performed a serial transfer evolution experiment with two treatments (Figure 1) and 14 transfers at a subinhibitory BAC concentration (4 μg mL^−1^). Phenotypic adaptions were assessed at the end of the evolution experiment by determining survival after 2 h in the presence of BAC, the growth rate, the lag time and the MIC for BAC of the evolved populations (Figure 3).

Single evolved lines either showed increased or decreased cell number upon BAC exposure. While the ancestor showed an average reduction of viable cells by 40% (Figure 2C), no killing after 2 h BAC exposure was observed for three of six lines of the survival‐only treatment (S) and six of six lines for the corresponding controls (Ctrl S) (Figure 3A). In contrast, no killing was observed for two of six lines for the survival and growth treatment (SG) and three of six lines for the corresponding controls (Ctrl SG) (Figure 3A). Statistical analysis between the evolutionary treatments and their corresponding controls showed that there was no significant difference in the surviving fraction in response to the treatment with BAC (Mann–Whitney *U* test, *p* < 0.05; Figure 3A). This indicates that mutations that are not specific to BAC exposure confer increased survival in single evolutionary lines and that selection for survival is generally a weak driver for differences between both evolutionary treatments (S and SG) in our experiment.

Furthermore, growth curves of the evolved lines in the presence of BAC were characterized, using a microplate reader to determine optical density (OD) over time. The data showed that the evolutionary treatments affected the growth rate (ANOVA *p* = 1.7e^−86^; Figure 3B). However, post hoc analysis showed a significantly different growth rate only in part (~58%) of the replicate evolutionary lines of the treatments and their corresponding controls as compared to the ancestor (Bonferroni post hoc test, *p* < 0.05). The evolved treatments were not significantly different from their respective controls (Mann–Whitney *U* test, *p* > 0.05). The altered growth rates in the presence of BAC in treatment and control populations in comparison with the ancestor suggest that the changes of growth rates are underpinned by unspecific adaptation to the general conditions of the evolution experiment rather than to the specific treatments with BAC.

In addition, the data showed that the evolutionary treatments resulted in an earlier increase in OD values during growth in the presence of BAC as compared to the ancestor (ANOVA, *p* = 1.5e^−13^; Figure 3C). These increased OD values were likely associated with earlier onset of growth (i.e., shorter lag time). Five of six of the evolved lines in the evolutionary treatment in the survival‐only (S), and survival and growth (SG) treatment had significantly earlier increase in OD than the ancestor (Bonferroni post hoc test, *p* < 0.05). While the OD of the ancestor started to increase at 8.7 h in the presence of BAC, the evolved treatment lines that were significantly different exhibited increased ODs at around 7.2–7.8 h. The lines that evolved with exposure to BAC had significantly earlier OD increases in comparison with their corresponding controls evolved without BAC (Mann–Whitney *U* test, *p* < 0.05). This shows that the presence of BAC during evolution selected for earlier increases in OD. Increases in OD can be explained by faster growth, increased survival, or by a shorter lag time. However, the first two mechanisms were not significantly different across all replicates (Figure 3A,B), suggesting that shorter lag times are overall the strongest driver of earlier OD increases.

Importantly, the populations that underwent selection for survival and growth acquired the ability to grow in the presence of higher BAC concentrations (Mann–Whitney *U* test, *p* = 0.007; Figure 3D). While the populations selected for growth and survival had a 1.1–1.6‐fold increase in MIC relative to the ancestor, the MIC of populations selected for survival‐only remained unchanged (0.9–1.1‐fold difference). This suggests that selection for growth in the presence of BAC underlies the increase in MIC. Selection for survival‐only does not affect the MIC, underscoring the difference in the evolutionary treatments.

Taken together, the phenotypic analysis of the responses to BAC showed that the differences between the evolutionary treatments are linked to the applied selection pressure. While evolutionary treatments with selection for survival‐only, and for survival and growth selected for a shorter lag time, only selection for survival and growth resulted in populations that were able to grow at elevated BAC levels. However, while differences in lag time and MIC of BAC are significant, the observed effects on the phenotype are relatively weak which might be explained by the applied selection pressure and the short duration of the evolution experiment.

### 3.3 Consequences of Selection in the Presence of BAC for Antibiotic Susceptibility

We investigated whether the distinct exposure to BAC in the two different evolutionary treatments differentially affected antibiotic susceptibility. To this end, we measured MICs to a range of selected antibiotics that have different mode of actions. The data show that there is overall a decreased antibiotic susceptibility of populations coming from both evolutionary treatments (Figure 3D). For ampicillin, we determined a significant 1.3‐fold MIC increase for the survival and growth treatment (SG; One‐Sample Wilcoxon signed rank test, *p* = 0.02) and significant 1.5‐fold MIC increase for the survival‐only treatment (S; One‐Sample Wilcoxon signed rank test, *p* = 0.031). For colistin and gentamycin, we observed a heterogeneous adaptation regarding antibiotic susceptibility that occurred in both evolutionary treatments (One‐Sample Wilcoxon signed rank test *p* > 0.05 for all conditions). Specifically, about half of the populations exhibited a 1.5–2‐fold MIC increase, while 6 of 24 populations did not show a change in MIC relative to the ancestor. For ciprofloxacin, the data showed no significant increase in MIC for populations exposed to the survival‐only treatment (S; One‐Sample Wilcoxon signed rank test, *p* = 0.37), while the survival and growth treatment showed a significant 1.5‐fold increase (SG; One‐Sample Wilcoxon signed rank test, *p* = 0.037). Taken together, there was a low effect of the BAC evolutionary treatments on the antibiotic susceptibility and weak parallelism between replicate lineages for specific antibiotics. In addition, adaptation of antibiotic susceptibility during BAC exposure occurs upon selection for survival‐only as well as for survival and growth depending on the type of antibiotic. For example, the difference in susceptibility to ciprofloxacin between the evolutionary treatments indicates that both selective drivers (growth or survival) during BAC exposure can differentially affect antibiotic susceptibility of BAC‐adapted lineages.

### 3.4 Evolved Genotypes Are Linked to BAC‐Mediated Selection Pressure

**FIGURE 4:**
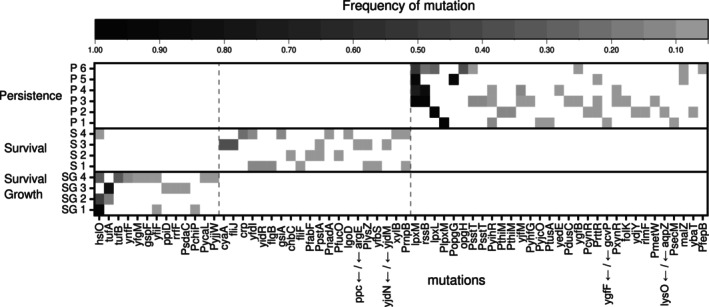
Mutated genes and promotor regions in response to different evolutionary treatments with exposure to benzalkonium chloride. Heatmap of the mutated genes, promotor regions (genes and promotor, depicted with P on x‐axis, with 5% frequency cut off) and mutations in intergenic regions (both genes divided by/surrounding the intergenic region, depicted with arrows indicating location, with 5% cutoff) in the evolved *E. coli* lines clustered to the treatments: Selection for survival and growth (SG), selection for survival‐only (S), and selection for persistence (P; data taken from (Nordholt et al. 2021)). The intensity scale depicts the frequency of mutations in the specific loci. The specific mutations are listed in Data S2.

Next, we asked whether the differences in BAC‐mediated selection pressure and the associated phenotypes are underpinned by differences in the acquired mutations of the evolved genotypes. To this end, genotypic adaptions were assessed at the end of the evolution experiment by sequencing the replicate populations of both evolved lines (S and SG) and their corresponding controls (Figure 4, Data S2). After identifying the mutations, the mutated genes that occur in the control lines were removed from the list of mutations considered for further analysis of the experimental treatments. In addition, we incorporated an additional dataset already published by our group into the analysis, in which replicate lines were evolved over 12 treatment cycles that consisted of exposure to a lethal BAC concentration above the MIC for 15 min, followed by growth in the absence of BAC (Figure S1) (Nordholt et al. 2021). This treatment was shown to select for increased tolerance in a persister subpopulation and thus was designated as selection for persistence (P). The same, M9 preadapted ancestor *E. coli* strain was used for all evolution experiments (S, SG and P). Sequence analysis was conducted by setting a threshold of 5% occurrence of mutations in the total reads of each sample. Intragenic regions or genes that were mutated in the control lines were not considered for the analysis of the mutations that occurred in the evolutionary lines treated with BAC. Thus, only BAC and treatment‐specific mutations were further compared by categorizing mutations into intra‐ and intergenic (Figure S2), clustering mutated genes according to treatment (Figure 4) and by categorizing mutated genes using the BioCyc GO terms (main and subcategory) (Figure S3).

The analysis showed 44 mutations in six sequenced lineages selected for persistence, 24 mutations in four sequenced lineages selected for survival‐only and 16 mutations in four sequenced lineages selected for survival and growth (Figure S2). The degree of fixation of mutations in specific genes or promoter regions was highest in the treatment selecting for persistence (five of six lines had 100% fixation for at least one gene). The treatment selecting for survival and growth had intermediate rates of fixation (one of four lines had 100% fixation for mutations in at least one gene) and the treatment selecting for survival‐only had the lowest fixation (the highest fixation rate of 38% for mutations of one gene was reached by one of four lines) (Figure 4). Overall, there were more intergenic than intragenic mutations in the treatment selecting for persistence (P), while for the other treatments (S and SG) more intragenic than intergenic mutations are present (Figure S2). Most of the intergenic mutations occurred in the promoter regions across treatments (S = 80%, SG = 100%,  P = 82%). This indicates that adaptation to BAC exposure was driven by adaptation of gene expression.

The mutated genes that were present in at least 5% of the total population were clustered according to treatment. This analysis showed that a distinct set of mutations in specific genes and promoter regions were associated to each evolutionary treatment (Figure 4). All mutations identified in BAC‐treated and control lineages are provided in Data S2. In the selection for persistence treatment, the mutated genes with the highest frequency and parallelism are associated with the membrane (*lpxM* and *lpxL*) and the stress response (*rssB*). Selection in the survival‐only treatment showed low parallelism with only two replicate lines having a mutation in the same gene (*yfdL*). In addition to mutations in *yfdL*, mutated genes were associated with regulation (*cyaA*) and cellular structure (flagella *fliJ*). In the selection for survival and growth treatment, the mutated genes with the highest parallelism are associated with protein folding stress and translation elongation (*hslO* and *tufA*).

Next, we clustered mutated genes and promoter regions according to treatment and functional categories (Figure S3). This analysis showed that mutations in the treatment for survival and growth and the treatment for survival‐only do not occur in shared functional categories. In contrast, the treatment for persistence shared mutations in functional categories with both evolutionary treatments (S and SG). The treatment selecting for survival and growth and the treatment selecting for persistence shared mutations in genes and promotor regions which are associated with metabolism (GO category metabolism of other compounds), cell processes (adaptations) and information transfer (protein‐related). The treatment selecting for survival‐only and the treatment selecting for persistence shared mutations in the functional categories in regulation (type of regulation) and metabolism (synthesis of macromolecules).

Taken together, we found differences between the treatments regarding the number of mutations, the level of fixation, the parallelism between replicate lines, and the identity of mutated genes and their functional categories. These differences indicate that each of the three evolutionary treatments is associated to a distinct selection pressure leading to different evolved adaptations.

## 4 Discussion

We determined the effects of different selection regimes in serial transfer evolution experiments using subinhibitory BAC concentrations with two distinct treatments (i) selection for survival‐only and (ii) selection for survival and growth. The used subinhibitory BAC concentration reduced the growth rate of the *E. coli* ancestor and caused initial killing upon exposure and thus its presence exerts a selection pressure on growth and survival. We hypothesized that the distinct selection pressures in both treatments select for distinct adaptation mechanisms.

Indeed, the characterization of the evolved phenotypes and genotypes revealed treatment‐specific adaptations. Distinct and significant phenotypic adaptations between BAC‐exposed and nonexposed controls were apparent in lag times and growth at increased BAC or antibiotic concentrations (Figure 3C,D). Treatment‐specific genotypic adaptations were apparent in clustering of mutated genes according to the selection pressure (Figure 4). Shorter lag times were selected in both treatments, both of which were exposed to BAC in the lag phase. Thus, shorter lag times might be related to selection during this phase. In contrast, the presence of BAC during the growth phase was required to select for growth at increased BAC concentrations (Figure 3D). This is in agreement with previous observations, showing that selection for survival‐only did not lead to increased MICs (Nordholt et al. 2021).

Overall, the determined phenotypes and genotypes showed signs of relatively weak adaptations and adaptations towards the general experimental conditions that were unspecific to BAC. BAC unspecific adaptations were apparent because the effects on the evolved growth rates (Figure 3B) and survival fractions (Figure 3A) of the controls were comparable to the evolutionary lines exposed to BAC. Weak, but partly significant, adaptation to BAC was apparent as relatively small differences in susceptibility to BAC or antibiotics (Figure 3D) and as a low degree of fixation and parallelism of mutated genes as compared to selection for persistence (Figure 4). This indicates that adaptive laboratory evolution experiments with subinhibitory BAC concentrations exert a low selection pressure and thus require a high number of generations to establish treatment‐specific adaptations that are fixed within the population. Therefore, we argue that standard serial transfer evolution experiments are not an ideal approach to select for increased growth upon BAC exposure (Jia, Lu, and Zhu 2022). One reason for low selection pressures is the narrow selective window of BAC (Figure 2A) which does not allow to dynamically fine tune the exposure to concentrations that decrease the growth rate but do not lead to full growth inhibition or eradication of the population. In turn, selecting for growth‐only at increased concentrations may be facilitated by experimental evolution approaches that allow to continuously adjust the selection pressure by increasing exposure concentrations in morbidostat continuous culture systems or on gradient plates (Baym et al. 2016; Carsenti‐Etesse et al. 1999; Toprak et al. 2011). These approaches would also allow to select on growth‐only, a treatment with a selection pressure that could not be established with the serial transfer approach used in this study.

The low degrees of fixation and parallelism could be explained by the narrow population bottleneck (i.e., dilution of the culture between transfer cycles) applied in our experiments. Wide bottlenecks (e.g., 0.1) decrease genetic drift and increase the effect of fitness gains of mutations, and therefore result in faster fixation of beneficial mutations and potentially fitter genotypes that are restricted to local fitness optima (leading to a higher degree of parallelism). We applied relatively narrow bottlenecks (0.01 for selection of persistence in Nordholt et al. 2021 and 0.001 in this study) and thereby likely increased the stochastic sampling of the fitness space, allowing genotypes to reach multiple fitness optima different from the nearest, local fitness optimum.

To our knowledge, this is the first attempt to disentangle the distinct selection pressures on survival and growth of an antimicrobial in serial transfer evolution experiments. Our data show that during serial transfer evolution experiments these different selection pressures are present. This observation could be also valid for other bactericidal antimicrobials including biocides and antibiotics (Wakamoto et al. 2013). Experimental evolution with antibiotics has shown that tolerance is a stepping stone for the evolution of resistance (Fridman et al. 2014; Levin‐Reisman et al. 2017; Van den Bergh et al. 2016). Tolerance may be selected by increased survival during the initial exposure phase with subinhibitory concentrations. Stress response during the initial exposure phase is known to be mediated by phenotypic heterogeneity and thus this heterogeneity may serve as basis for the evolution of increased survival (Nordholt et al. 2021; Van den Bergh et al. 2016). While we observed heterogenous killing in the ancestor (Figure 2C) under our experimental conditions, we did not investigate the basis of this heterogeneity in detail. However, since phenotypic heterogeneity has been shown to be involved in the evolution of tolerance to BAC (Nordholt et al. 2021), it is likely that it also plays a role under our experimental conditions. It should be noted that the level of phenotypic heterogeneity in tolerance might depend on the conditions of the pre‐culture (medium, growth phase, and time in stationary phase) and on the conditions during the lethal exposure (medium and cell density).

An apparent phenotypic adaptation was that evolved lines showed reduced lag times (Figure 3C). One possible consequence of the evolved shorter lag times might be the increase of the biomass‐to‐BAC ratio to counteract BAC toxicity as an extension of the well‐known inoculum effect. Decreasing toxicity by increasing growth rates have been shown to dilute antibiotics inside single cells, enhancing phenotypic tolerance (Łapińska et al. 2022).

An alternative explanation for the evolution of shorter lag time is that surviving cells evolve mechanisms that counteract accumulation of cellular damage, allowing them to resume growth earlier than nonevolved cells. Those mechanisms have been shown previously to be related to increased efflux (Buffet‐Bataillon et al. 2012; Merchel Piovesan Pereira, Wang, and Tagkopoulos 2021; Pagedar, Singh, and Batish 2012) or reduced uptake by increasing outer membrane charge (Nordholt et al. 2021). In addition, increasing chaperon activity could minimize cellular damage. Accordingly, the treatment selecting for survival and growth displayed mutations in the redox‐regulated chaperon Hsp33 encoded by the gene *hslO* (Graf et al. 2004), and the highly abundant and essential regulator of the polypeptide chain elongation cycle during the translation process, the elongation factor EF‐Tu encoded by the gene *tufA* (Thompson, Dix, and Karim 1986). The activity of *hslO* is regulated by redox state upon oxidative stress conditions (Winter et al. 2005), while overexpression of *tufA* has been shown to rescue the activity of oxidative stress‐sensitive *hslO* knock‐outs in *Vibrio cholerae* (Wholey and Jakob 2012). Similarly, the treatment selecting for survival‐only had a loss‐of‐function mutation in *cyaA*, which has been reported to be involved in pan tolerance to antibiotics and biocides by reducing ROS formation (Zeng et al. 2022). The gene *cyaA* encodes for adenylate cyclase, which synthesizes the second messenger 3′,5′‐cyclic adenosine monophosphate (cAMP) being involved in regulation of sugar metabolism. A loss‐of‐function mutation in *cyaA* decreases metabolic activity that leads to ROS formation in cells residing in the lag phase reducing damage of biomass (Zeng et al. 2022). Thus, the loss‐of‐function mutation in *cyaA* evolved in the survival‐only treatment may facilitate increased survival by altering the metabolic state of cells leading to decreased ROS formation. In contrast, chaperones like *hslO* enable constant removal of damaged biomass to allow growth in the presence of BAC, explaining its occurrence in the survival and growth treatment. In summary, the evolved phenotypes and genotypes allow to hypothesize that the two distinct treatments led to the evolution of distinct mechanisms to cope with BAC induced redox stress. If BAC is present during the growth phase, it selects for active redox regulation and removal of proteins damaged by oxidative stress. In contrast, the accumulation of redox species is avoided if BAC is present in the lag phase only.

The findings of this study have implications for the design and interpretation of adaptive laboratory evolution experiments that aim to assess the risks of adaptive mechanisms upon exposure to antimicrobials. The design of the evolution experiment needs to be aligned to the expected selection pressure exerted by the antimicrobial on the microorganisms. For example, if one aims to determine adaptive mechanisms to a lethal dose of a disinfectant, one should expose cells to a relevant lethal dose thereby selecting for increased survival (Nordholt et al. 2021). An evolution experiment with sublethal concentrations might not be suitable to determine specific adaptive mechanisms to lethal doses because our experiment showed that growth in the presence of the disinfectant selected for distinct mechanisms. Growth in the presence of an active substance is unexpected during the process of disinfection. In contrast, if one aims to determine adaptive mechanisms to a diluted disinfectant in the environment or to a preservative aimed to inhibit growth, one should determine whether the relevant concentration reduces the growth rate or leads to partial killing. If the relevant concentration does not induce partial killing, the concentrations in the evolution experiment should be adjusted to a nonlethal dose because our experiment showed that evolution for survival selects for distinct adaptive mechanisms. In conclusion, our work highlights the importance of fine tuning the selection pressures present in adaptive laboratory evolution experiments to the relevant exposure scenarios.

## Conflicts of Interest

The authors declare no conflicts of interest.

## Supporting information


Data S1.

Data S2.

## Data Availability

Phenotypic data (Figures 2 and 3) for this study are available at the Dryad Digital Repository:https://doi.org/10.5061/dryad.2jm63xszx. Sequencing data has been deposited in the sequence read achieve (SRA) at NCBI under the accession number BioProject PRJNA1074740.
