# Supplementary material for: Differential Selection for Survival and for Growth in Adaptive Laboratory Evolution Experiments With Benzalkonium Chloride
Source: Evol Appl. 2024 Oct 12;17(10):e70017. doi: 10.1111/eva.70017 (PMC11470201; doi:10.1111/eva.70017)
Supplement: Supplementary file 1 — Data S1. [file EVA-17-e70017-s002.docx]

Supplementary information

**Differential selection for survival and for growth in adaptive laboratory evolution experiments with benzalkonium chloride**

**Fig S1. BAC exposure during the treatment and growth phase in the different evolution experiments analyzed for genotypic difference in Figure 4.**

Relative concentrations of BAC applied in the evolutionary treatments during the exposure (black bars) and growth phase (grey bars). The y-axis shows the treatment concentration divided by the minimum inhibitory concentration (MIC) measured for the *E. coli* ancestor. The dotted line indicates the level of the MIC of the *E. coli* ancestor. The evolutionary treatment conducted in the serial transfer evolution experiments had two treatments with subinhibitory BAC concentrations, either selecting for survival and growth (SG) or survival only (S). In both treatments BAC is present at subinhibitory concentrations during the exposure phase (4 h). Only in the SG treatment a prolonged exposure (22 h) during the growth phase to subinhibitory BAC concentrations is given. P indicates a serial transfer evolution experiment conducted in a previously published study (Nordholt et al., 2021), which selected for an increase in an initially present tolerant sub-population; i.e. for increased persistence. In this experiment, BAC was applied at lethal concentrations in the exposure phase (15 min) and highly diluted (1:100) BAC concentrations were present during the growth phase.

**Fig S2. Number of mutations in the intra- or intergenic area for the different evolutionary treatments as analyzed for the genotypic data shown in Figure 4.**

Number of mutated loci (intragenic and intergenic) with a 5% frequency cut-off in sequencing reads. SG – selection for survival and growth; S – selection for survival only; P – selection for persistence (Nordholt et al., 2021). Note that S and SG include data from 4 and P from 6 lineages evolved in parallel.

Unclassified

**extrachromosomal**

**regulation**

**metabolism**

**cell processes**

**information transfer**

**transport**

**cell structure**

SG

S

P

**Fig S3. Mutations clustered by functions in the different evolutionary treatments as analyzed for the genotypic data shown in Figure 4.**

Mutations occurring in the different treatments with a 10 % frequency cut-off in sequencing reads were clustered and categorized using BIOCYC GO Terms (main and sub-category). The color code depicts the main category. The dotted lines divide the different evolutionary treatments. SG – selection for survival and growth; S – selection for survival only; P – selection for persistence (Nordholt et al., 2021). Data are included in supplementary table 2.

**Table S1.** Absolute MIC values of the *E. coli* MG1655 ancestor and the evolved lineages for benzalkonium chloride (BAC) and different antibiotics and given in µg mL^-1^

|  | **BAC^1^** | **colistin^2^** | **gentamicin^2^** | **ciprofloxacin^2^** | **ampicillin^2^** |
| --- | --- | --- | --- | --- | --- |
| ancestor | 6.4 | 0.125 | 0.19 | 0.007 | 2 |
| Survival-only lineage 1 | 9 | 0.125 | 0.158 | 0.006 | 2.5 |
| Survival-only lineage 2 | 9 | 0.19 | 0.19 | 0.008 | 3 |
| Survival-only lineage 3 | 8 | 0.19 | 0.19 | 0.008 | 3 |
| Survival-only lineage 4 | 10 | 0.25 | 0.38 | 0.008 | 3 |
| Survival-only lineage 5 | 7 | 0.25 | 0.38 | 0.008 | 4 |
| Survival-only lineage 6 | 9 | 0.25 | 0.25 | 0.008 | 3 |
|  |  |  |  |  |  |
| ancestor | 6.4 | 0.125 | 0.22 | 0.008 | 3 |
| Survival-growth lineage 1 | 6 | 0.158 | 0.38 | 0.008 | 4 |
| Survival-growth lineage 2 | 7 | 0.19 | 0.38 | 0.012 | 4 |
| Survival-growth lineage 3 | 6 | 0.19 | 0.5 | 0.012 | 4 |
| Survival-growth lineage 4 | 6 | 0.158 | 0.315 | 0.012 | 4 |
| Survival-growth lineage 5 | 6.7 | 0.19 | 0.25 | 0.012 | 4 |
| Survival-growth lineage 6 | 7 | 0.125 | 0.188 | 0.012 | 4 |

^1^ determined by microbroth dilution

^2^ determined by E-test
